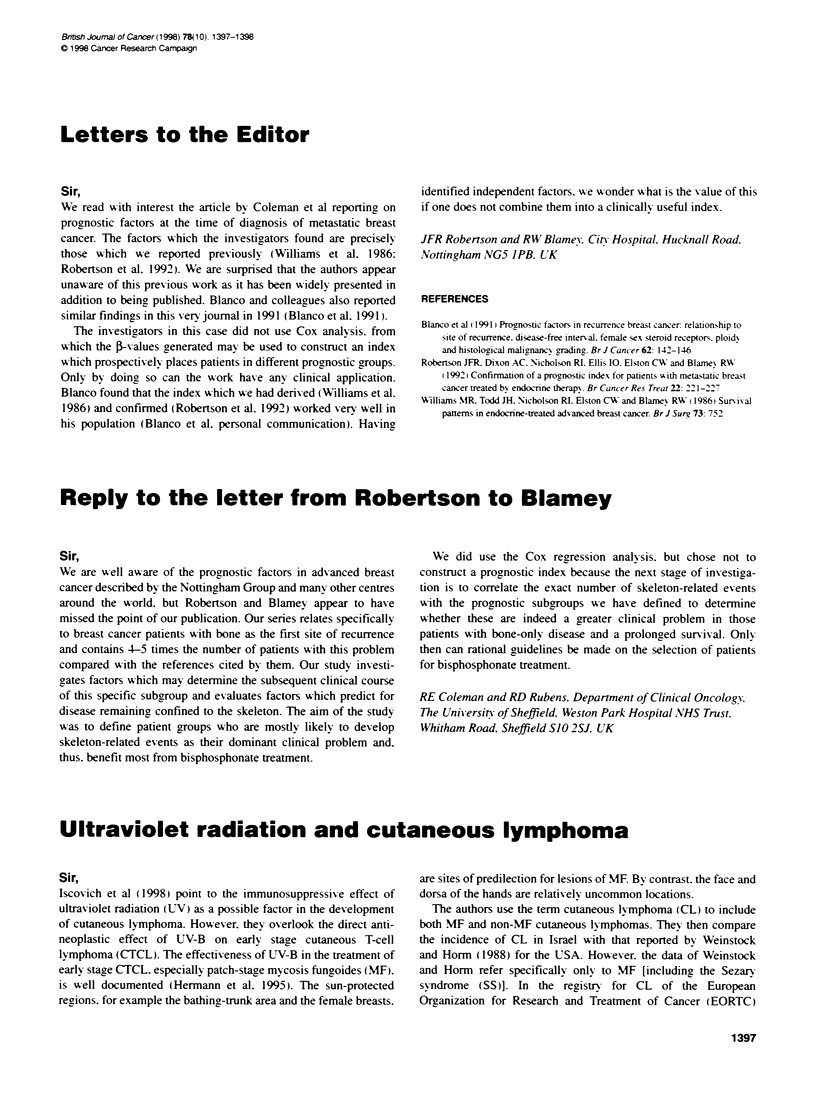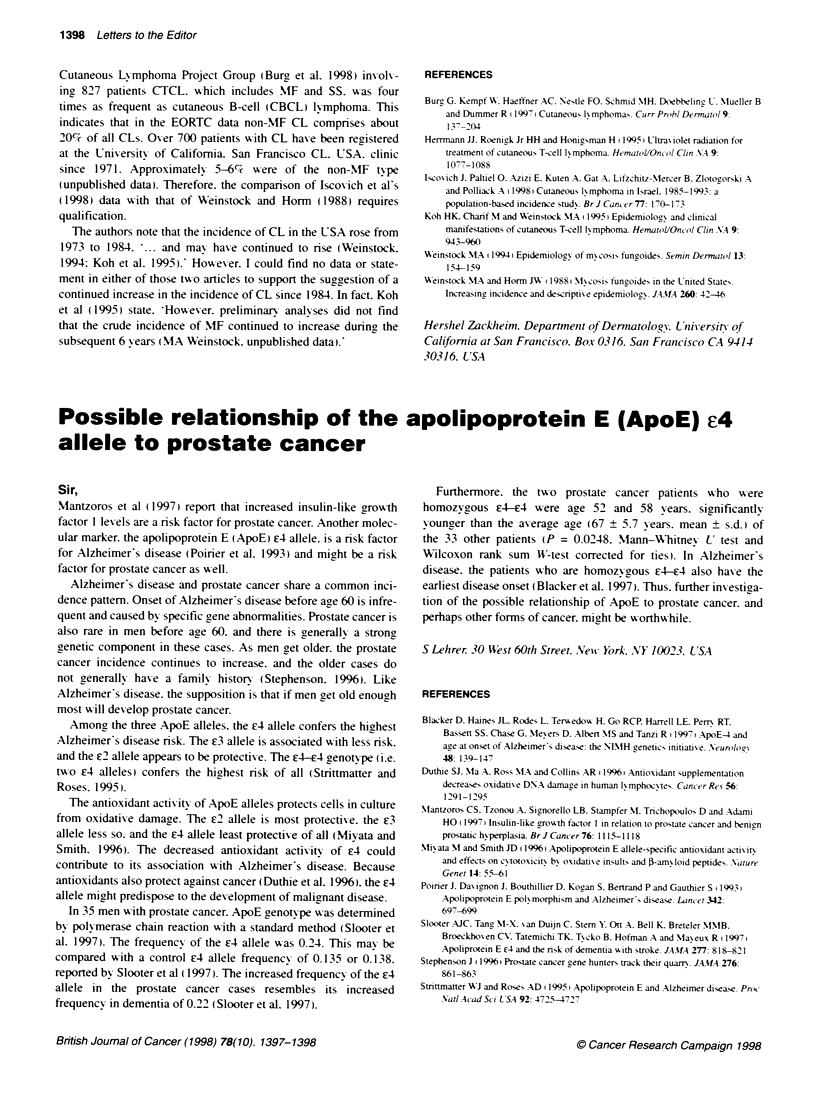# Ultraviolet radiation and cutaneous lymphoma.

**DOI:** 10.1038/bjc.1998.694

**Published:** 1998-11

**Authors:** H. Zackheim


### Ultraviolet radiation and cutaneous lymphoma

**Sir,**

Iscovich et al (1998) point to the immunosuppressive effect of ultraviolet radiation (UV) as a possible factor in the development of cutaneous lymphoma. However, they overlook the direct anti-neoplastic effect of UV-B on early stage cutaneous T-cell lymphoma (CTCL). The effectiveness of UV-B in the treatment of early stage CTCL, especially patch-stage mycosis fungoides (MF), is well documented (Hermann et al, 1995). The sun-protected regions, for example the bathing-trunk area and the female breasts, are sites of predilection for lesions of MF. By contrast, the face and dorsa of the hands are relatively uncommon locations.

The authors use the term cutaneous lymphoma (CL) to include both MF and non-MF cutaneous lymphomas. They then compare the incidence of CL in Israel with that reported by Weinstock and Horm (1988) for the USA. However, the data of Weinstock and Horm refer specifically only to MF [including the Sezary syndrome (SS)]. In the registry for CL of the European Organization for Research and Treatment of Cancer (EORTC)

Cutaneous Lymphoma Project Group (Burg et al. 1998) involving 827 patients CTCL, which includes MF and SS, was four times as frequent as cutaneous B-cell (CBCL) lymphoma. This indicates that in the EORTC data non-MF CL comprises about 20% of all CLs. Over 700 patients with CL have been registered at the University of California, San Francisco CL, USA, clinic since 1971. Approximately 5–6% were of the non-MF type (unpublished data). Therefore, the comparison of Iscovich et al's (1998) data with that of Weinstock and Horm (1988) requires qualification.

The authors note that the incidence of CL in the USA rose from 1973 to 1984, '... and may have continued to rise (Weinstock, 1994; Koh et al, 1995).' However, I could find no data or statement in either of those two articles to support the suggestion of a continued increase in the incidence of CL since 1984. In fact, Koh et al (1995) state, 'However, preliminary analyses did not find that the crude incidence of MF continued to increase during the subsequent 6 years (MA Weinstock, unpublished data).'

**REFERENCES**

Burg G, Kempf W, Haeffner AC, Nestle FO, Schmid MH, Doebbeling U, Mueller B and Dummer R (1997) Cutaneous lymphomas. *Curr Probl Dermatol* **9**: 137–204

Herrmann JJ, Roenigk Jr HH and Honigsman H (1995) Ultraviolet radiation for treatment of cutaneous T-cell lymphoma. *Hematol/Oncol Clin NA* **9**: 1077–1088

Iscovich J, Paltiel O, Azizi E, Kuten A, Gat A, Lifzchitz-Mercer B, Zlotogorski A and Polliack A (1998) Cutaneous lymphoma in Israel, 1985–1993: a population-based incidence study. *Br J Cancer* **77**: 170–173

Koh HK, Charif M and Weinstock MA (1995) Epidemiology and clinical manifestations of cutaneous T-cell lymphoma. *Hematol/Oncol Clin NA* **9**: 943–960

Weinstock MA (1994) Epidemiology of mycosis fungoides. *Semin Dermatol* **13**: 154–159

Weinstock MA and Horm JW (1988) Mycosis fungoides in the United States. Increasing incidence and descriptive epidemiology. *JAMA* **260**: 42–46

*Hershel Zackheim, Department of Dermatology, University of California at San Francisco, Box 0316, San Francisco CA 94143 0316, USA*